# Supplementary material for: Predicting visual function by interpreting a neuronal wiring diagram
Source: Nature. 2024 Oct 2;634(8032):113–23. doi: 10.1038/s41586-024-07953-5 (PMC11446822; doi:10.1038/s41586-024-07953-5)
Supplement: Supplementary file 6 — CRF and ERF predictions for individual TmY4 and TmY9 cells. Analogous to Supplementary Data 3, but for TmY target types. Shown are the top four monosynaptic pathways, the strongest pathway passing through each of the top ten intermediary types (ranking from Extended Data Fig. 7), and the trisynaptic pathway Tm1–TmY–Dm3–TmY (see the section entitled Prediction of spatial normalization). [file 41586_2024_7953_MOESM6_ESM.zip › DataS4/TmY9qΓèÑ/720575940628078346.pdf]

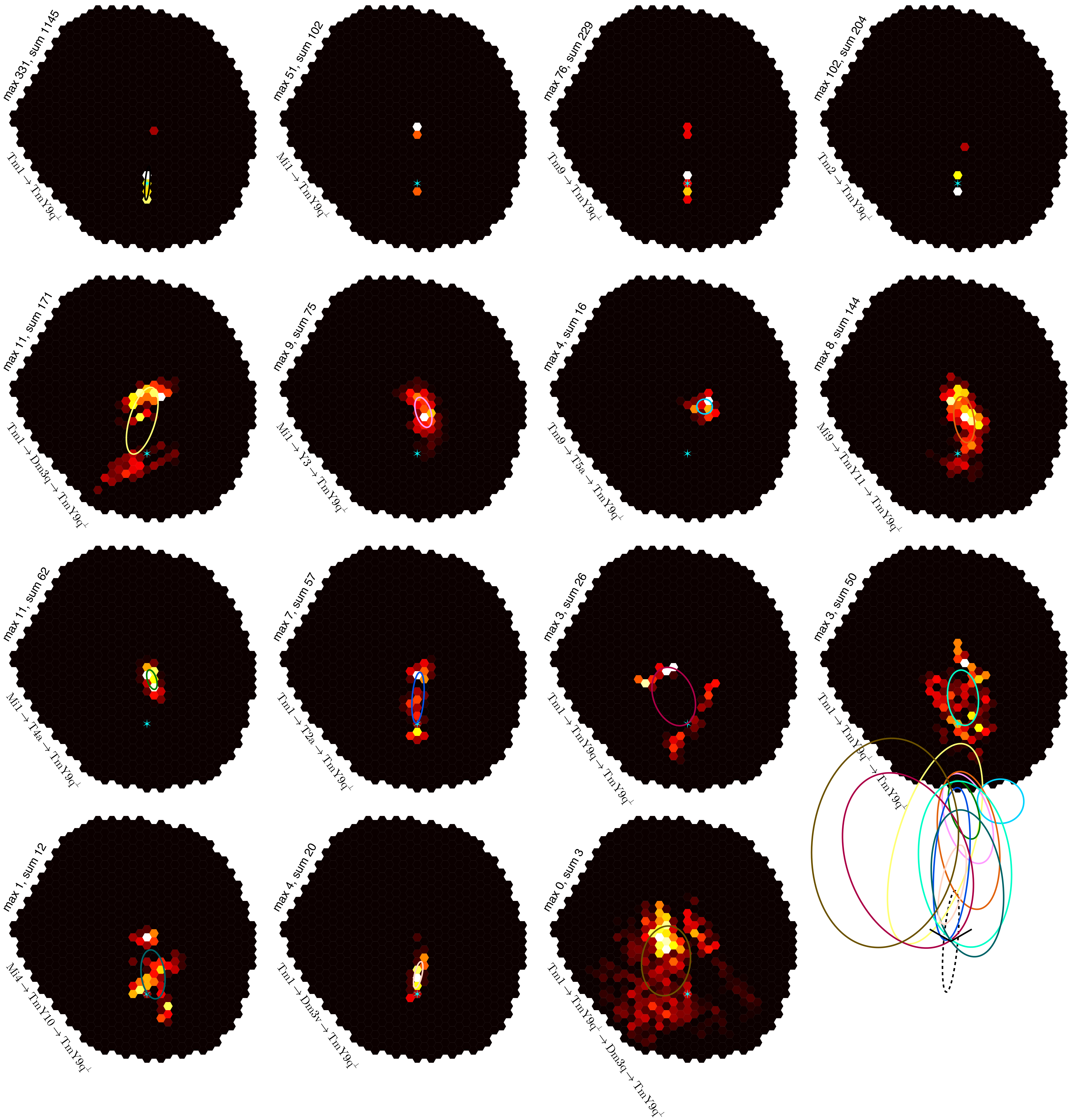

max 331, sum 1145
Tm1 → TmY9q⊥
max 51, sum 102
Mi1 → TmY9q⊥
max 76, sum 229
Tm9 → TmY9q⊥
max 102, sum 204
Tm2 → TmY9q⊥
max 11, sum 171
Tm1 → Dm3q → TmY9q⊥
max 9, sum 75
Mi1 → Y3 → TmY9q⊥
max 4, sum 16
Tm9 → T5a → TmY9q⊥
max 8, sum 144
Mi9 → TmY11 → TmY9q⊥
max 11, sum 62
Mi1 → T4a → TmY9q⊥
max 7, sum 57
Tm1 → T2a → TmY9q⊥
max 3, sum 26
Tm1 → TmY9q → TmY9q⊥
max 3, sum 50
Tm1 → TmY9q⊥ → TmY9q⊥
max 1, sum 12
Mi4 → TmY10 → TmY9q⊥
max 4, sum 20
Tm1 → Dm3v → TmY9q⊥
max 0, sum 3
Tm1 → TmY9q⊥ → Dm3q → TmY9q⊥
